# Elevated Maspin Expression Is Associated with Better Overall Survival in Esophageal Squamous Cell Carcinoma (ESCC)

**DOI:** 10.1371/journal.pone.0063581

**Published:** 2013-05-22

**Authors:** Yang Wang, Shijie Sheng, Jianzhi Zhang, Sijana Dzinic, Shaolei Li, Fang Fang, Nan Wu, Qingfeng Zheng, Yue Yang

**Affiliations:** 1 Thoracic Surgery, Key Laboratory of Carcinogenesis and Translational Research Ministry of Education, Peking University School of Oncology, Beijing Cancer Hospital & Institute, Beijing, China; 2 Department of Pathology, Wayne State University School of Medicine, Tumor and Microenvironment Program of Barbara Ann Karmanos Cancer Institute, Detroit, Michigan, United States of America; Southern Illinois University School of Medicine, United States of America

## Abstract

Tumor suppressor maspin is a differentially regulated gene in the progression of many types of cancer. While the biological function of maspin in blocking tumor invasion and metastasis is consistent with the loss of maspin expression at the late stage of tumor progression, the differential expression and the biological significance of maspin in early stage of tumor progression appear to be complex and remain to be elucidated. In the current study, we examined the expression of maspin in 84 esophageal squamous cell carcinoma (ESCC) cases (stages I–III) and 55 non-tumor adjacent esophageal tissue specimens by immunohistochemical (IHC) staining. The correlation of maspin with clinicopathological parameters was analyzed. Compared to normal esophageal squamous tissue where 80% (47/55) of the cases expressed maspin at a low to moderate level, all ESCC specimens (100% (84/84)) were positive for maspin expression at a moderate to high level. ESCC with low or moderate maspin expression had significantly shorter postoperative survival rates compared to those that had high maspin expression (p<0.001). Since the correlation of maspin with ESCC histology and the correlation of maspin with ESCC prognosis seem to be at odds, we further investigated the biological function of maspin in ESCC using the established ESCC cell lines. The expression of maspin in five human esophageal squamous cancer cell lines (T12, E450, KYSE150, EC109, and KYSE510) was examined by the Western blot. ESCC cell line KYSE510 that did not express maspin and was stably transfected by maspin cDNA or an empty vector. The resulting transfected cells were characterized in vitro. Maspin expression significantly inhibited cell proliferation, motility and matrigel invasion. Taken together, our data suggest that the transient up-regulation of maspin in the early development of ESCC may be a defense mechanism against further transition towards more malignant phenotypes, ultimately slowing down ESCC tumor progression.

## Introduction

Esophageal carcinoma, one of the most aggressive carcinomas of the gastrointestinal tract, is the eighth most common cause of cancer-related death worldwide [1], [2]. The two main subtypes of esophageal carcinoma are adenocarcinoma and squamous cell carcinoma (ESCC), while the most commonly diagnosed esophageal cancer in China and other Asian countries is ESCC [3], [4]. Despite the rapid advancement in combined chemotherapy and radiation therapy for ESCC, the average 5-year overall survival has remained steady at 10–20% [2], [5], [6]. Unfortunately, the prediction of clinical prognosis of patients with ESCC based on conventional pathological variables, such as the tumor size, tumor grade, and the tumor stage is highly empirical [7]–[9]. Specific biomarkers that are mechanistically involved in the progression of ESCC may significantly improve the accuracy of the prediction of patients’ survival. To this end, it is important to note that many of the molecular markers that are associated with specific pathological grades (diagnosis) have failed to serve as prognostic markers. It is well appreciated that tumor progression is a continuum of dynamic molecular and cellular changes. The link of a molecular profile with a phenotype may not reflect whether the former is driver or a passenger of the latter, and may not predict whether this association is consequential for further tumor progression. Some molecular changes may be suppressive steps that may eventually give way to the predominant oncogenic changes. The capacity of tumor cells to turn on tumor suppressive mechanisms, even though transitory, may translate into delayed tumor progression, and prolonged patients’ survival.

Maspin is an epithelial-specific tumor suppressor that is differentially regulated during tumor progression. It is a member of the serine protease inhibitor (serpin) superfamily [10] but with functions that are deviant from those of classical serine protease-inhibiting serpins. Accumulated evidence suggests that maspin may play a key role in the maintenance of epithelial homeostasis by blocking serine protease-like enzymes such as the zymogen form of urokinase-type plasminogen activator [11], [12] and histone deacetylase 1 [13]. Consistent with its anti-invasion and anti-metastasis properties, maspin expression is found to be down regulated in the progression of many types of cancer at the step of tumor invasion and metastasis.

As a tumor suppressor, maspin is not immediately down-regulated in the early development of cancer. In fact, accumulated evidence demonstrates a transient up-regulation of maspin in non-invasive cancer of breast [14], [15], ovary [16], and pancreas [17], [18]. In adenocarcinomas, this up-regulation of maspin is also associated with its translocation from the nucleus to the cytoplasm. In squamous cell carcinomas, however, maspin is always distributed to both nucleus and cytoplasm. The biological significance of these distinct differential patterns is a subject of current investigation. Based on our earlier studies, the translocation of maspin from the nucleus to the cytoplasm in early stage adenocarcinoma correlates with significantly better survival of lung cancer [19]. On the other hand, elevated maspin expression in early stage squamous cell carcinoma both, nuclear and cytosolic, correlates with significantly better survival of oral squamous cell carcinoma [20]. These data suggest that the prognosis of cancer subtypes may be distinctly stratified based on maspin differential expression.

In this paper, we report the first clinical evidence that maspin was significantly elevated in a subpopulation of stage I-III ESCC specimens. The level of maspin expression correlated with better overall survival of ESCC patients. We further investigated the biological function of maspin using established ESCC cell lines and showed an inhibitory effect of maspin on cell proliferation, motility, and invasion. These data suggest that ESCC with an ability to up-regulate maspin may be protected against further malignant progression.

## Materials and Methods

### Tissue Specimens

This research involves 84 archived formalin-fixed and paraffin-embedded human stage I–III esophageal squamous cell carcinoma (ESCC) tissue specimens, from patients who were eligible for and underwent surgical resection between 2003 and 2007 at the Department of Surgery, Beijing Cancer Hospital (China). In addition, tumor-adjacent normal tissue specimens were collected from 55 of these patients. Prior to the tissue collection, the clinical protocol was approved by the ethics review board of Peking University Health Sciences Center. Informed written consents were obtained from the patients. The consents were saved as scanned PDF files, and saved in patients’ records. The samples were de-identified to the research group. The 84 patients included 64 men and 20 women with a median age of 58 years (ranging from 43 to 73 years).

### Immunohistochemical (IHC) Staining

Tissue sections on slides of 5 µm thickness were subjected to hematoxylin and eosin (H&E) staining, and IHC staining of maspin as described previously [16]. Monoclonal antibody against maspin (clone G167–70; Pharmingen/BD Bioscience, San Diego, CA) was diluted 100-fold. The horse-reddish peroxidase (HRP) conjugated secondary antibody (Dako Cytomation, Cambridgeshire, UK) was detected by chromogenic reaction of HRP. For negative controls, the primary antibody was omitted. The tissue sections were examined and scored independently by the two pathologists who had no prior knowledge of the study aim or design. Maspin expression was semi-quantitatively evaluated by the percentage of maspin positive cells and the intensity of maspin staining on the scale of 0–3. The percentage of positive cells was categorically scored as following: 0 points (maspin positive in 0–5% of cells); 2 points (maspin positive in 6–50% of cells); 3 points (maspin positive in >50% of cells). The staining intensity was categorically scored as follows: 1 point: negative or weak staining; 2 points: moderate staining; 3 points: strong staining. The overall maspin expression (OMS) was calculated as the sum of the percentage category points and the intensity category points in each case. Tumors were categorized into four groups: negative: ≤5% of cells stained, regardless of intensity; weak expression (OMS: 0–2 points); moderate expression (OMS: 3–4 points); and strong expression (OMS: 5–6 points). Maspin sub-cellular pattern of nuclear staining and cytoplasmic staining were assessed semi-quantitatively on the basis of the percentage of positive cells, as described previously [21]. Maspin nuclear immunoreactivity was defined as stronger nuclear staining than cytoplasmic staining in at least 10% of tumor cells. Maspin cytoplasmic immunoreactivity was defined as stronger cytoplasmic staining than nuclear staining in at least 10% of tumor cells.

### Cell Lines and Cell Culture

Five human ESCC cell lines (KYSE-510, KYSE-150, T12, E450 and EC-109) were purchased from Institute of Basic Medical Sciences Chinese Academy of Medical Sciences’ cell culture center (Beijing, China). The cells were maintained at 37°C and 5% CO_2_ in RPMI-1640 medium (GIBCO, US) supplemented with 10% heat-inactivated fetal bovine serum (FBS) and 1% penicillin-streptomycin.

### Western Blotting

Cells were lysed with 1× RIPA buffer (Beyotime Institute of Biotechnology, Nantong, China) containing 25 µg/mL leupeptin (Sigma Chemical Co., St. Louis, MO) and 10 µg/mL aprotinin (Sigma Chemical Co.). Cells were removed from the dishes by cell scraping. The samples were then subjected to three cycles of freeze-thaw and centrifuged at 12,000 rpm for 20 min. The protein concentration of the samples was determined using a bicinchoninic acid Protein Assay Reagent kit, and whole cell lysates were analyzed by 10% SDS-PAGE and transferred onto a polyvinylidene fluoride (PVDF) membrane. The membrane was blocked in 5% skim milk for 1 hr at room temperature (RT) and then probed with primary antibodies against maspin (1∶500 diluted) and α-actin (from SIGMA, 1∶10,000 diluted), respectively. The HRP-conjugated anti-mouse secondary antibody was used at 1∶2,500 dilutions. The bound secondary antibody was detected by chemiluminescence reaction (Millipore, Bedford, USA) and visualized by radiography.

### Stable Transfection

Sequence-verified maspin cDNA cloned into a vector for expression in mammalian cells [22] was used to transfect approximately 60% confluent KYSE-510 using the Lipofectamine™ 2000 kit (Invitrogen, Carlsbad, CA). The empty vector DNA was used in parallel transfection as a negative control. For clonal selection, 24 hrs after transfection, G418 was added to the culture medium at the concentration of 400 µg/mL. The cells were maintained in G418-containing medium for the next 4 weeks until individual clones were selected. The selected clones were subsequently maintained in the medium containing 200 µg/mL of G418.

### Cell Proliferation Assay

To determine the effect of maspin on cell proliferation, cells with and without maspin were seeded into 96-well plate at a density of 4×10^3^/well/200 µL in the maintenance medium. Viable cells were quantified at 24, 48, 72 and 96 hours (h) after the seeding by the chromogenic 3-[4,5-dimethylthiazol-2-yl]-2,5-diphenyl-tetrazoliumbromide (MTT) assay according to the manufacturer’s instruction (Sigma, St. Louis, US). Each assay was performed in triplicates and repeated 3 times.

### Colony Formation Assay

Cells seeded in 6-well plates at a density of 400 cells/well/medium volume, were allowed to grow for 10 days to form colonies. The cells were washed twice with PBS, and treated with Giemsa for 10 min, and then photographed with a digital camera (OLYMPUS, SP350). The number of colonies and the number of cells in each colony was counted under the microscopy. The colonies which had more than 100 cells were defined as big colonies.

### Wound-healing Assay

The cells were added to six-well plates, allowed to form confluent monolayers and were serum starved overnight. An artificial wound was created in the cell monolayer with a sterile plastic 200 µL micropipette tip to generate one homogeneous wound in each well. After wounding, the culture medium was removed, and cells were washed at least twice to eliminate detached cells. Wound closure was photographed at 0, 6, 12, and 24 h after wounding. Images of cells from the same field were acquired at the indicated time points, using an inverted microscope equipped with a digital camera. The number of cells in each colony was counted under microscope. Each measurement was performed in triplicate.

### Matrigel Invasion Assay

Invasion assay was performed using 8 µm PET pore size membrane coated with Matrigel (24-well, BD Biosciences, Bedford, MA). Cells were seeded at 2×10^5^ cells per 500 µL of growth medium on the Matrigel-coated membrane. The bottom wells were filled with the maintenance culture medium, and the chambers were incubated at 37°C in a humidified 5% CO_2_. After 24 h, the Matrigel and non-invading cells in the upper chamber were removed by scraping. The cells on the bottom side of the membrane (invading cells) were stained with 1% crystal violet and counted under the microscope. Each experiment was performed in triplicate.

### Statistical Analysis

Clinicopathological factors were analyzed separately using the chi-square test. The Kaplan-Meier method was used to estimate the patient survival and the log-rank test was used to determine the statistical significance. The associations between discrete variables were assessed using the chi-square test. All data were expressed as the mean ± standard deviation. A *p*-value of less than 0.05 was considered to be statistically significant. Statistical calculations were performed using the Statistical Package for Social Sciences software version 11.0 (SPSS11.0).

## Results

### Differential Maspin Expression in Human ESCC and Normal Adjacent Tissues

**Figure 1:**
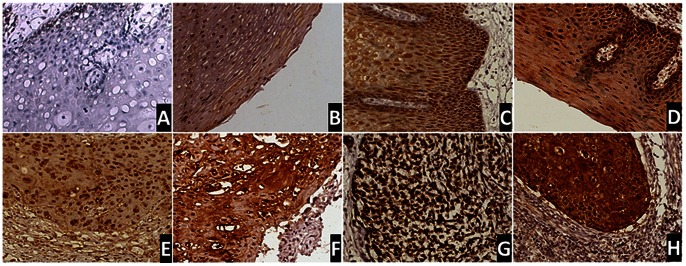
Representative IHC of maspin in matched normal and esophageal squamous cell carcinoma tissues. Top: (A) normal esophageal tissue with negative, (B) weak, (C) moderate, and (D) strong maspin staining. Bottom: (E) ESCC with weak overall maspin expression, (F) moderate overall maspin staining, (G) overall strong maspin staining, which is distributed more to the nucleus, and (H) overall strong maspin staining, which is distributed more to the cytoplasm. x200.

To investigate whether the level or subcellular localization of maspin helps predict the survival of ESCC patients, ESCC tissues were collected from 84 stage I-III patients who were eligible for and underwent surgical resection between 2003 and 2007. These patients were followed up for at least 5 years. IHC was performed to examine the expression of maspin in these tumor specimens, as well as 55 tumor-adjacent normal tissues, and the level of maspin expression was semi-quantified using the method described in the Materials and Methods section. Representative results are shown in **Figure 1**. As summarized in **Table 1**, among 55 cases of normal esophageal tissues, the positive immunoreactivity was associated with 85% of cases (47/55). The 8 patients had no maspin immunoreactivity, 37 cases had weak maspin staining, and 10 cases were associated with strong maspin staining. In contrast, all 84 ESCC specimens showed positive maspin staining. 33 tumor specimens were associated with weak maspin staining, whereas 51 cases had strong maspin staining. Overall, as compared to the corresponding normal esophageal tissues, ESCC tissues exhibited stronger maspin IHC signals and a higher percentage of maspin-strong cells (*p*<0.001).

**Table 1:** Differential Maspin Expression in ESCC and Matched Normal Tissues.

	Negative OMS 0–1	Weak/Moderate OMS 2–4	Strong OMS 5–6	*P* Value
ESCC tissues	0	33	51	**<0.001**
Normal tissues	8	37	10	

Based on the overall maspin expression, all the tumor specimens can be divided by the total level of maspin expression (low/moderate vs. strong), the level of nuclear maspin (weak/moderate vs. strong), and the level of cytoplasmic maspin (weak/moderate vs. strong). The stratification of these different groups with clinicopatholoigcal variables was evaluated. As summarized in **Table 2**, there was no significant difference between the groups with respect to sex, age, pathologic grade and tumor stage. In addition, we also analyzed the association between subcellular localization of maspin and clinicopathological variables. Even though not statistically significant, we observed the following trends: poorly differentiated tumors were associated with weaker nuclear maspin staining and stronger cytoplasmic maspin staining. In addition, the lymph node metastasis was associated with a higher level of cytoplasmic maspin staining as compared to lymph node negative patients.

**Table 2:** Correlation of ESCC Clinicopathological Features with Maspin Expression and Subcellular Localization.

Variable	Cases (n = 84)	Maspin Expression		*P* Value	Nuclear Maspin		*P* Value	Cytoplasmic Maspin		*P* Value
		Weak	Strong		Weak	Strong		Weak	Strong	
***Sex***										
Male	64	26 (40%)	38 (60%)	0.653	28 (43%)	36 (57%)	0.767	36(57%)	28 (43)	0.922
Female	20	7 (35%)	13 (65%)		8 (40%)	12 (60%)		11 (55%)	9 (45%)	
***Age***										
<60	42	16 (37%)	26 (63%)	0.823	17 (40%)	25 (60%)	0.659	25 (60%)	17 (40%)	0.510
≥60	42	17 (40%)	25 (60%)		19 (45%)	23 (55%)		22 (52%)	20 (48%)	
***Differentiation***										
Well	19	8 (43%)	11 (57%)	0.706	9 (47%)	10 (53%)	0.475	8 (41%)	11 (69%)	0.383
Moderate	43	18 (42%)	25 (58%)		20 (46%)	23 (54%)		26 (60%)	17 (40%)	
Poor	22	7 (32%)	15 (68%)		7 (31%)	15 (69%)		13 (61%)	9 (39%)	
***Lymph node metastasis***										
Negative	33	11 (33%)	22 (67%)	0.369	14 (43%)	19 (57%)	0.949	18 (58%)	15 (42%)	0.834
Positive	51	22 (43%)	29 (56%)		22 (40%)	29 (60%)		29 (60%)	22 (40%)	
***Pathologic stage***										
I+II	40	13 (32%)	27 (65%)	0.225	16 (40%)	24 (60%)	0.614	19 (48%)	21 (52%)	0.137
III	44	20 (45%)	24 (54%)		20 (45%)	24 (55%)		28 (63%)	16 (37%)	

### Maspin Expression Correlates with Better Overall Postoperative Survival

**Figure 2:**
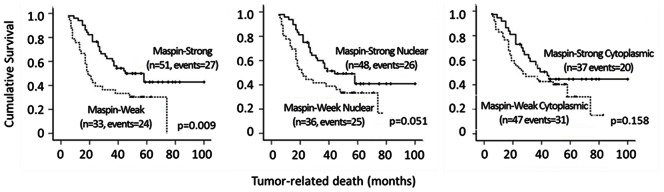
Kaplan-Meier survival curves of ESCC. (A) Cases stratified based on the overall maspin expression, (B) levels of maspin staining in the nucleus, and (C) the levels of maspin expression in the cytoplasm.

Our cohort of ESCC patients were followed up for at least 5 years, as of March 2012, with a median survival of 36.5 months. In order to test if maspin expression is associated with increased or decreased patient survival, patients were classified into two groups, those with strong maspin expression (51 cases, OMS 5–6) or those with weak and moderate maspin expression (33 cases, OMS 2–4). The corresponding median survival time for these two groups were 45±11.1 months and 19±2.9 months, respectively, demonstrating that stronger maspin expression is associated with significantly increased patient survival and favorable prognosis. (**Figure 2A**, log-rank, *p* = 0.009). Since previous reports in breast [23] and lung cancer [19] for example, suggest that nuclear maspin is associated with better overall patient survival, we also investigated whether maspin subcellular localization correlated with the overall survival. For this purpose, the patients were classified into two corresponding groups: predominantly nuclear expression or predominantly cytoplasmic expression patients. Although the *p* value did not reach a significant difference, stronger expression of maspin in the nucleus was still associated with a more favorable patient prognosis (44±9.5 months vs. 21±4.5 months; *p* = 0.051) (**Figures 2B and 2C**). In general, maspin expression level correlated with less tumor local invasion. We also observed a trend for the negative correlation between maspin expression and lymph node metastasis (**Table 2**). In addition, reduced nuclear maspin and increased cytoplasmic maspin were associated with lymph node metastasis. Although these data did not reach statistical significance, in part limited by the number of patients, they are consistent with the notion that the mortality of these ESCC patients result primarily from metastasis.

### Maspin Expressed in ESCC Cell Lines is Tumor Suppressive

**Figure 3:**
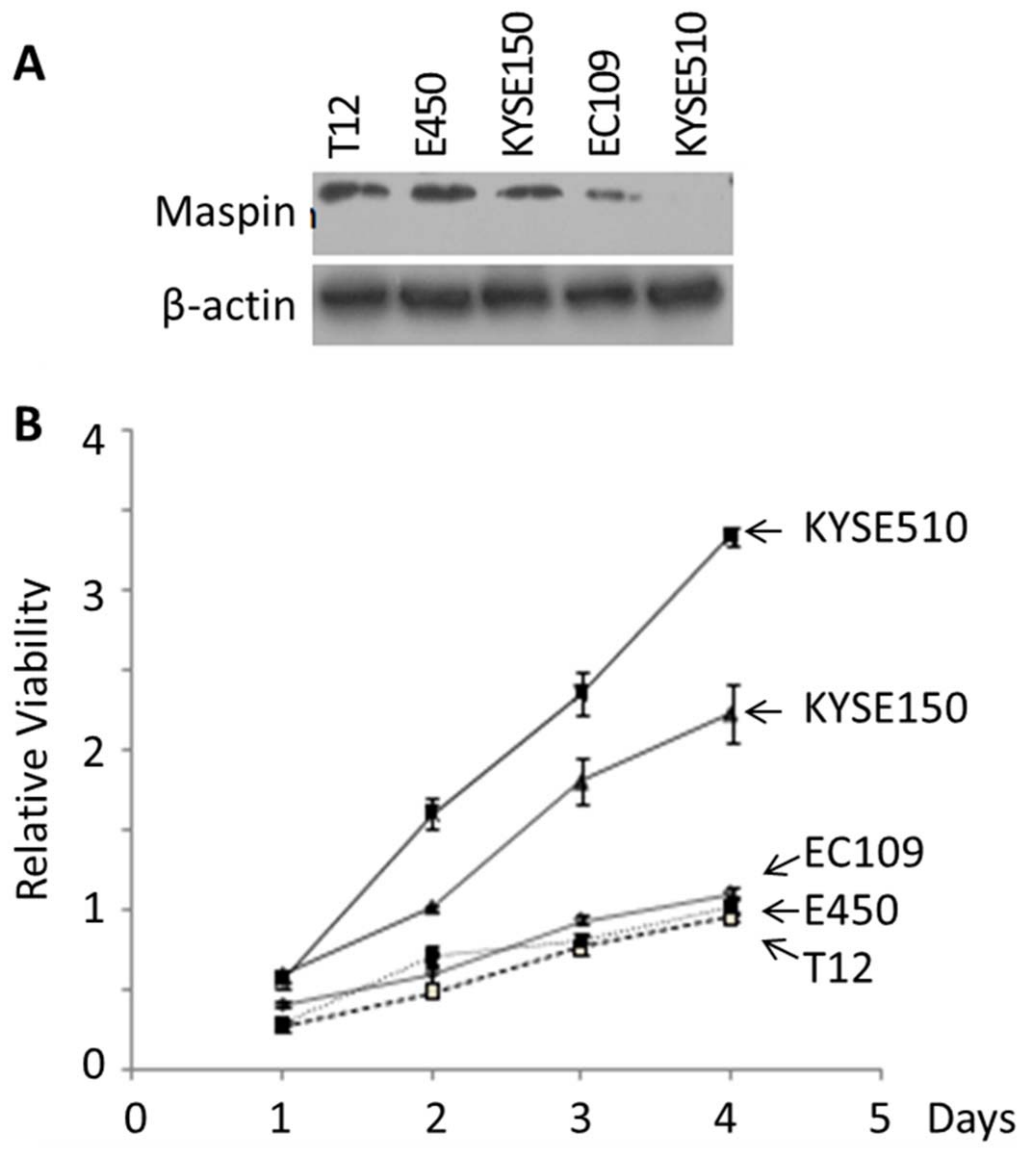
The correlation of maspin expression in established human ESCC cell lines with lower rates of proliferation *in vitro*. (A) Western blotting of maspin in the indicated ESCC cell lines. Twenty-five micrograms of total lysate protein were loaded in each lane. Western blotting of the same membrane for house-keeping β-actin was used to assess the loading variation. (B) MTT assay of the proliferation of ESCC cell lines. The data at each time point represent the average of three independent repeats. The error bars represent the standard deviation.

Established ESCC cell lines offer a valuable experimental model to study the progression and underlying molecular mechanisms. Similar to the observation with early stage ESCC specimens, the level of maspin was not significantly altered in ESCC cell lines that are only weakly or moderately aggressive (T12, E450, KYSE150, and EC109) [24], as judged by Western blotting (**Figure 3A**). However, ESCC cell line KYSE510 that was significantly more invasive and grew at a faster rate had lost maspin expression (**Figure 3B**).

**Figure 4:**
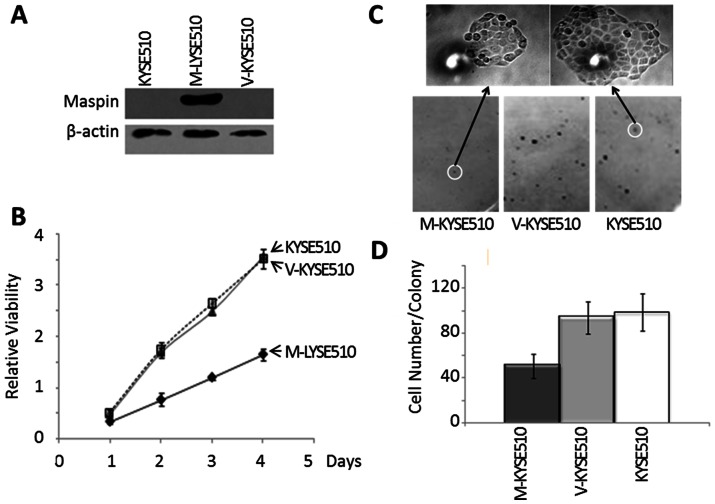
Characterization of stably transfected KYSE510 cell lines. (A) Western blotting of maspin and housekeeping protein β-actin in the total lysates of parental KYSE510, M-KYSE510, and V-KYSE510 cells. (B) MTT assay of the proliferation of parental KYSE510, M-KYSE510, and V-KYSE510 cells, cultured in the maintenance media. (C) Representative staining of single cell-derived colonies (bottom) and the magnified image of the highlighted colonies (top) from the colony formation assay. (D) Quantification of colonies with more than >100 cells/colony based on counting under microscope in the colony formation assay. Data represent the average of three independent repeats. Error bars represent the standard deviation. The difference between M-KYSE51 and V-KYSE510 (or parental KYSE510) was statistically significant (*p*<0.001).

To investigate the functional significance of maspin in ESCC, we took the advantage of KYSE510 cell line that did not express maspin and stably transfected the cells with maspin-encoding pCMV-Tag2-maspin vector or an empty vector pCMV-Tag2. The maspin expression in the resulting maspin transfected cells (M-KYSE510) and the mock transfected control (V-KYSE510) are shown in **Figure 4A**. As shown in **Figure 4B**, maspin expression correlated with decreased cell proliferation as judged by the MTT assay. Earlier, it was reported that maspin regulated cell attachment and detachment [13], [25]. To determine whether the effect of maspin expression on tumor growth was a result of altered colonization, cells were suspended and seeded at a low density in cell culture dish. The number of the single cell-derived colonies and number of cells per colony were evaluated under the microscope. Maspin expression did not significantly alter the colony forming ability, for the colony numbers for M-KYSE510, V-KYSE510 and the parental cells were not significantly different (data not shown). However, as shown in **Figure 4C**, the size of M-KYSE510 colonies was significantly smaller compared to those derived fromV-KYSE510 cell line. Consistently, the number of cells per colony was significantly lower than those for V-KYSE510 and parental cells. The number of colonies of M-KYSE510 with more than 100 cells was approximately a half of those of V-KYSE510 or parental KYSE510cells (*p<*0.01, **Figure 4D**).

**Figure 5:**
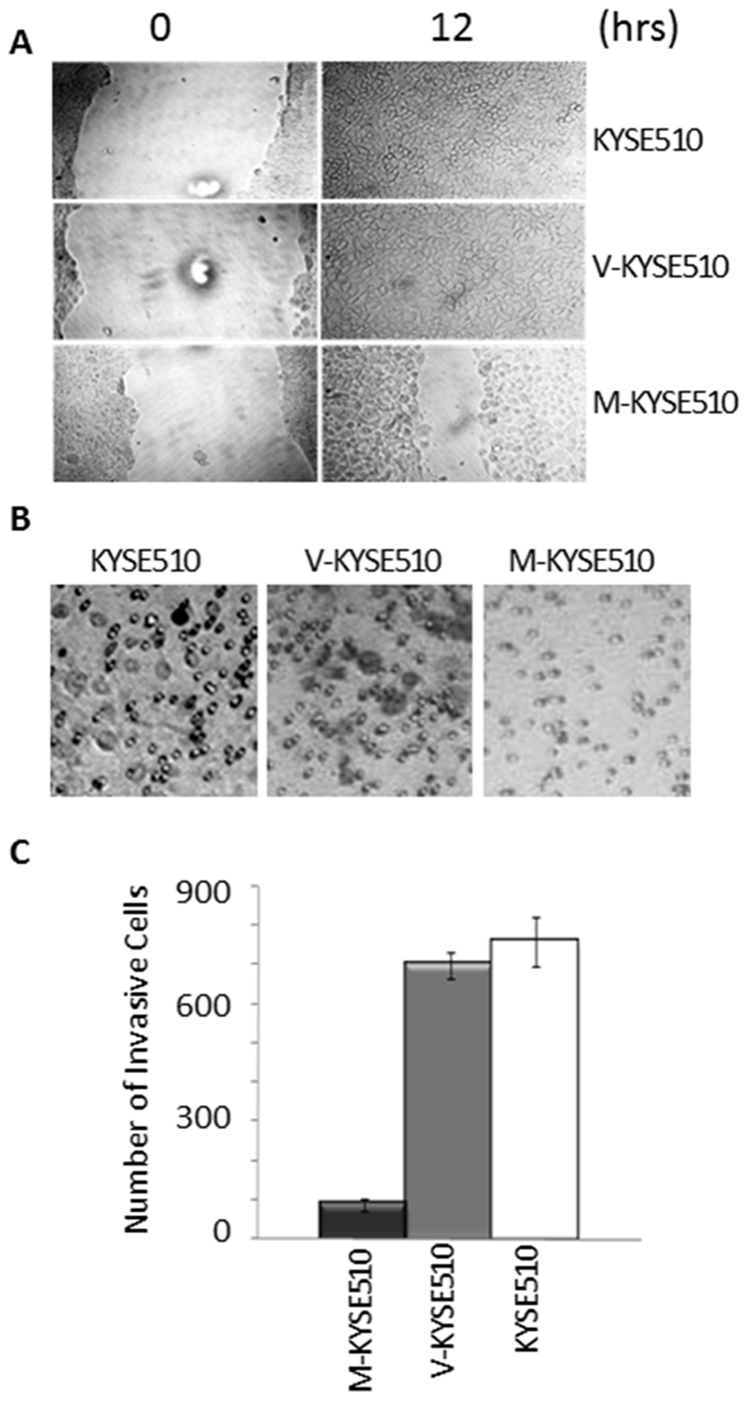
The effects of maspin expression on cell motility and invasion. (A) Representative microscopic images of post-wounding monolayer cell culture at the indicated time points. (B) Representative microscopic images of the underside of the transwell invasion assay membrane. (C) Quantification of the number of invasive cells based on the counting of the invading cells shown in (B). M-KYSE510 showed significantly lower numbers of invasive cells. Data represent the average of three independent repeats. Error bars represent the standard deviations.

To further investigate whether the presence of maspin in ESCC is a gain or loss of other functions in tumor progression, we examined the effect of maspin expression on tumor cell motility and invasion. As shown by the *in vitro* wound healing assay (**Figure 5A**), The M-KYSE-510 displayed a significantly attenuated rate of wound healing as compared to V-KYSE-510 or parental KYSE-510 cells. Moreover, as compared to V-KYSE-510 or parental KYSE-510 cells, M-KYSE-510 exhibited a significantly lower capacity to migrate through the Matrigel-coated transwell membrane in the *in vitro* invasion assay (*p*<0.01, **Figures 5B and 5C**).

Although the level of maspin correlated with the overall survival, the biological function and underlying molecular mechanisms of maspin may not be as simple. The effects of maspin may further depend on tumor microenvironments. In addition to reducing the proliferative activity and invasive potential, maspin may also prevent tumor angiogenesis through epigenetic regulation [26]–[30].

Earlier we have shown that maspin may directly inhibit cell surface-associated uPA to block tumor cell detachment [12], [26], [31], [32]. Data from the Hendrix Laboratory [33] and our laboratory [29] further suggest that maspin may down-regulate the expression of uPA. To test whether the effect of maspin on the motility and invasiveness of ESCC cells was, at least in part, due to its effect on uPA, we performed real-time PCR for uPA. Interestingly, the level of uPA in all the cell lines tested was low, irrespective to the levels of maspin (data not shown). Considering the differences between squamous cell carcinoma and adenocarcinoma, the underlying mechanism for the inhibitory effects of maspin on ESCC invasion may not be identical to that in carcinoma cells of breast and prostate origin. To date, there is no clinical consensus about uPA as a prognostic marker for ESCC.

## Discussion

The challenge for clinicians and oncologists in terms of patient personalized medicine and plan for treatment is that early stage tumors with similar histopathological features may subsequently display dramatically different outcome. In this paper, we described the first evidence that tumor suppressor maspin expression in early stage ESCC positively correlated with overall postoperative survival of patients. In light of our *in vitro* data that maspin inhibits tumor growth and blocks tumor invasion, several important observations with human specimens suggest a unique value of maspin as a molecular prognostic marker of ESCC.

**Figure 6:**
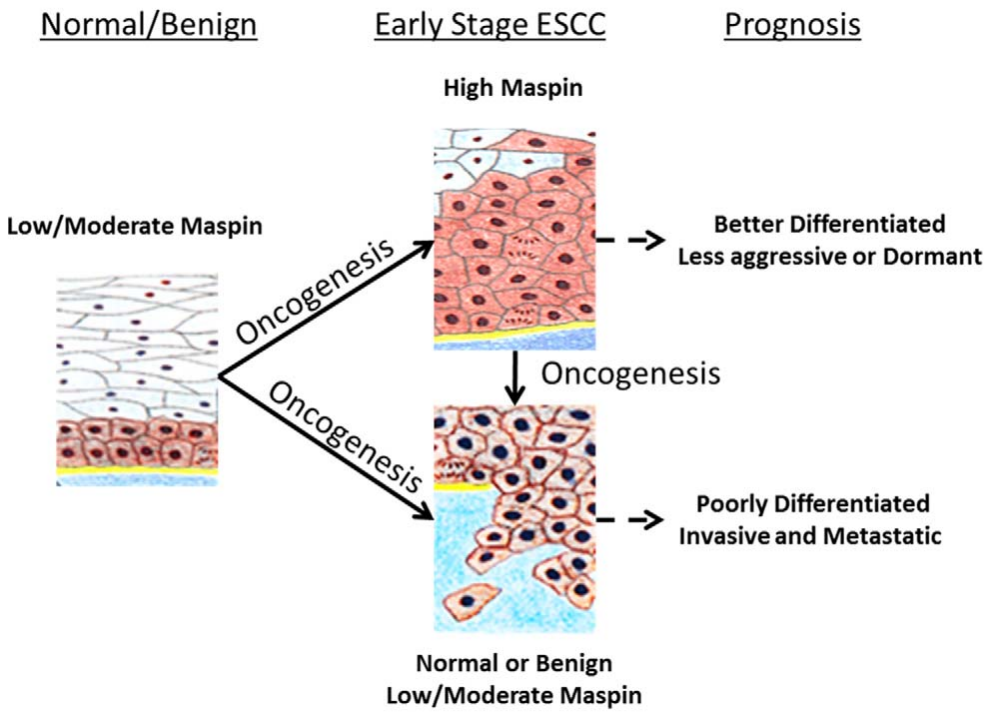
A hypothetical model for maspin as a prognostic marker of ESCC.

Overall, our data support a hypothetical model (**Figure 6**) that helps explain the correlation between maspin up-regulation and better overall survival of patients with ESCC. Based on this model, maspin is expressed in early stage ESCC to retain the epithelial homeostasis. In the absence of oncogenic changes, a basal level of maspin expression is maintained in normal or benign squamous epithelial cells to counter incidental stress and transformation insults. Upon the transformation and other oncogenic changes, the basal level of maspin expression may not be sufficient to counter-balance the biological effects of oncogenes. Those epithelial cells that are still capable of up-regulating maspin expression will remain better differentiated with low potential to invade and metastasize. The balance will shift towards more malignant phenotypes in those cells that are not capable of up-regulating maspin or would eventually lose maspin expression. In contrast to molecular markers whose differential expression patterns completely coincide with histopathological features, early stage ESCC cells expressing maspin at different levels may be histologically similar. The usefulness of maspin differential expression may not be to confirm pathological diagnosis. Rather, maspin may be uniquely useful as an independent marker to predict the course of the disease progression.

Maspin is an epithelial-specific protein. We did not detect specific maspin antigen by immunohistochemistry in stromal components in human ESCC specimens. Clinical studies to correlate maspin and tumor progression have been mostly conducted with adenocarcinoma, which is thought to be of glandular epithelial origin. Together with our current study, there are only two reports on how maspin expression correlates with the progression of squamous cell carcinoma, which derives from stratified squamous epithelial cells. The reported data with oral squamous cell carcinoma [20], [34] share the following important similarities with our current study with ESCC: (i) both studies were conducted with surgically resected early stage tumor specimens; (ii) in both cases, maspin protein was detected in almost all tumor cells, and was overexpressed in some tumor cells; (iii) maspin protein was detected in both the nucleus and cytoplasm; and (iv) the overall maspin expression levels correlated with better survival of the patients. In comparison, a distinct maspin differential expression patter is observed in adenocarcinoma. Studies with tissue specimens from breast [35], [36], prostate [37], and lung [19] adenocarcinoma showed that maspin is predominantly a nuclear protein in benign epithelial cells. Pre-neoplastic lesions and early stage carcinomas are commonly associated with elevated level of maspin, which is localized to both nucleus and cytoplasm [19], [35]. In invasive and metastatic carcinoma maspin expression is down-regulated or lost [38]–[40]. To our knowledge, maspin is the only molecular marker that displays distinct differential expression patterns in the progression of different subtypes of carcinoma.

It is noted that carcinogenesis and tumor progression are more driven by the loss of tumor suppressors than the activation of oncogenes [41]. Thus, tumor suppressor genes that are mechanistically involved in tumor progression may be more insightful molecular markers for diagnosis or prediction of prognosis. A paradigm based on the studies of classic tumor suppressor genes whose loss or mutation at the genetic level contributes to carcinogenesis and tumor progression would predict that tumor suppressors would be down-regulated as long as the oncogenesis is initiated [42]. Therefore, the tumor suppressive functions of maspin may seem to be at odds with the observation that maspin is actually transiently up-regulated in some cells that have already acquired the histopathologic features of tumor cells. To this end, it is important to point out that maspin is primarily regulated at the level of expression and trafficking. Although maspin is not as frequently mutated as some other well-known tumor suppressor genes such as p53 [43], a specific Ser^176^→Pro polymorphism has been identified [44] which seems to be frequent in gastric cancer and had reduced tumor suppressive potency as compared to the wild type maspin.

*In vitro* data from this study are in line with the consensus that maspin exerts multifaceted anti-tumor effects, inhibiting tumor growth, motility, invasion, and sensitizing tumor cells to drug-induced apoptosis. Maspin may be a nuclear, cytoplasmic, cell membrane-associated, as well as secreted molecule. The multifaceted biological activities of maspin may be coordinated by its molecular partnerships and subcellular localization [45]–[47]. To this end, the Sheng laboratory was the first to report that (i) secreted endogenous maspin binds and inhibits single-chain tissue type plasminogen activator (sc-tPA, a zymogen) that is bound to fibrin or fibrinogen [48], (ii) extracellular maspin specifically binds and inhibits pro-urokinase type plasminogen activator (pro-uPA, a zymogen) that is associated with cell surface-anchored uPA receptor (uPAR) [32] and (iii) intracellular maspin specifically interacts and inhibits histone deacetylase 1 (HDAC1) [13]. While the specific molecular mode of action of cytoplasmic maspin is under investigation in our lab, and earlier report suggested that cytoplasmic maspin regulates the Rho/Rac signaling network and block tumor cell motility [49]. It is likely that endogenous targets of maspin may be molecular targets for cancer therapy.
