# Correction: Elevated Maspin Expression Is Associated with Better Overall Survival in Esophageal Squamous Cell Carcinoma (ESCC)

**DOI:** 10.1371/journal.pone.0104715

**Published:** 2014-08-07

**Authors:** The PLOS ONE Staff

There is an error in Figure 4A. The authors have provided a corrected Figure 4 here.

**Figure 4:**
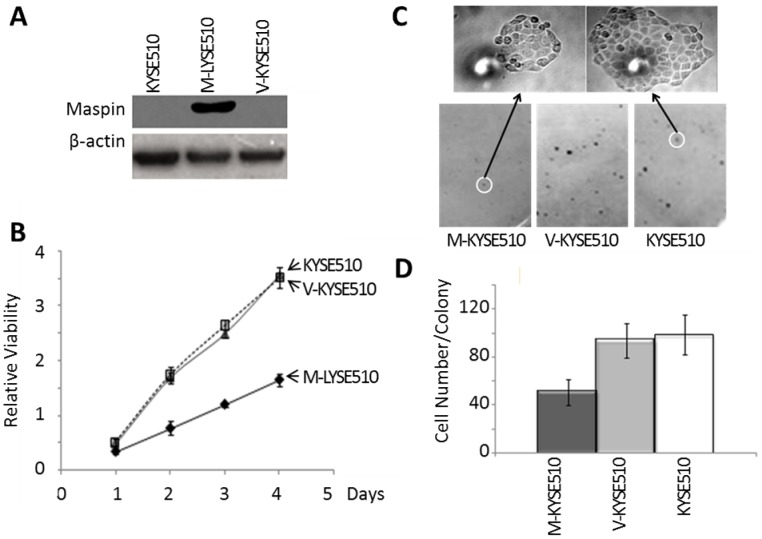
Characterization of stably transfected KYSE510 cell lines. (A) Western blotting of maspin and housekeeping protein β-actin in the total lysates of parental KYSE510, M-KYSE510, and V-KYSE510 cells. (B) MTT assay of the proliferation of parental KYSE510, M-KYSE510, and V-KYSE510 cells, cultured in the maintenance media. (C) Representative staining of single cell-derived colonies (bottom) and the magnified image of the highlighted colonies (top) from the colony formation assay. (D) Quantification of colonies with more than >100 cells/colony based on counting under microscope in the colony formation assay. Data represent the average of three independent repeats. Error bars represent the standard deviation. The difference between M-KYSE51 and V-KYSE510 (or parental KYSE510) was statistically significant (*p*<0.001).
